# Affective Computing in Serious Games for Physical Rehabilitation: Scoping Review

**DOI:** 10.2196/81344

**Published:** 2026-07-02

**Authors:** María del Pilar Beristain-Colorado, Patricia Batres-Mendoza, Erick Israel Guerra-Hernández, José Luis Cano-Pérez, Christian Perezcampos-Mayoral, Marciano Vargas-Treviño, Jaime Gutiérrez-Gutiérrez, Jorge Fernando Ambros-Antemate

**Affiliations:** 1 Laboratorio de Computación Centrada en el Humano aplicada a la salud y a la educación, Facultad de Sistemas Biológicos e Innovación Tecnológica, Universidad Autónoma Benito Juárez de Oaxaca, Av. Universidad S/N. Ex-Hacienda 5 Señores, Oaxaca de Juárez, Oaxaca, 68120, Mexico, 52 9515020712 ext 312; 2 Laboratorio de Inteligencia Artificial, Robótica y Control, Facultad de Sistemas Biológicos e Innovación Tecnológica, Universidad Autónoma Benito Juárez de Oaxaca, Oaxaca de Juárez, Oaxaca, Mexico; 3 Laboratorio de Fibras Ópticas y Sensores, Facultad de Sistemas Biológicos e Innovación Tecnológica, Universidad Autónoma Benito Juárez de Oaxaca, Oaxaca de Juárez, Oaxaca, Mexico; 4 Facultad de Sistemas Biológicos e Innovación Tecnológica, Universidad Autónoma Benito Juárez de Oaxaca, Oaxaca de Juárez, Oaxaca, Mexico

**Keywords:** affective computing, serious game, physical rehabilitation, rehabilitation technology, emotions

## Abstract

**Background:**

Serious games have become an alternative support for traditional physical therapy. However, many of these games do not address the emotional needs of patients. People with disabilities often experience emotions such as sadness, frustration, and even anger, which can create a barrier to their rehabilitation treatment.

**Objective:**

This review presents a comprehensive overview of technologies, techniques, and methods in affective computing as applied to serious games for physical rehabilitation and identifies key gaps to guide future research in this field.

**Methods:**

A scoping review was conducted following PRISMA (Preferred Reporting Items for Systematic Reviews and Meta-Analyses) guidelines, using the databases PubMed, ScienceDirect, IEEE Xplore, ACM Digital Library, PEDro, Springer, and Google Scholar.

**Results:**

The initial search yielded 5293 records, of which 9 papers met the inclusion criteria. Data were systematically extracted from these papers based on predefined research questions. Notably, engagement, tiredness, and pain were the most identified emotions, reported in 4 of 9 (50%) studies. Only 3 studies applied theoretical frameworks for emotion classification. Facial expression analysis and gesture recognition were the most frequently used affective computing techniques, yet only 2 studies implemented adaptive gameplay based on affective feedback.

**Conclusions:**

The integration of affective computing into serious games represents a promising approach for detecting affective states in patients undergoing rehabilitation. However, the limited number of primary studies, methodological limitations, and potential selection and reference-standard biases limit the reliability and generalizability of the current findings. Future research should prioritize rigorous multicenter designs, standardized evaluation protocols, and multidisciplinary collaboration. Developing these areas is essential to establishing clinical effectiveness.

## Introduction

### Overview of Serious Games for Physical Rehabilitation

Physical rehabilitation is a therapeutic approach aimed at improving the quality of life for individuals who have lost mobility in one or more body parts. Rehabilitation typically involves a series of repetitive exercises using the affected limb to gradually regain mobility. However, the repetitive nature of these exercises often becomes monotonous, leading to patient boredom and, in many cases, therapy discontinuation [1].

To address these challenges, alternative interventions such as serious games have been developed. Serious games are video games designed to be both entertaining and educational [2]. They have great potential for training, motivation, and entertainment. One of their key features is the ability to simulate reality in a virtual environment, enhancing user immersion and concentration. These types of games have been used in various fields, including health, education, politics, and engineering. Specifically, serious games designed for physical rehabilitation aim to provide a more motivating environment, enhance treatment adherence, and make repetitive exercises more engaging. They provide a different context within therapeutic interventions to help patients reacquire, recover, and maintain physical capabilities, thereby improving quality of life [3]. Applications include motor recovery following stroke, gait rehabilitation, Parkinson disease, multiple sclerosis, orthopedic recovery, and physical stimulation for individuals with cerebral palsy [4,5]. These interventions have gained considerable attention among researchers and health care professionals due to their potential to address complex rehabilitation needs through innovative technologies [6].

A wide range of serious games has been developed for physical rehabilitation [7,8], which have shown positive results compared to conventional rehabilitation therapies. For instance, these games enhance the quality, enjoyment, and effectiveness of rehabilitation by providing an engaging environment and varying levels of difficulty based on injury severity. They can also overcome logistical limitations by enabling patients to engage in therapy remotely, attending clinical sessions only when necessary [5,9]. However, literature reviews show that most existing platforms focus on motor metrics, such as range of motion and kinematics, and often overlook the patients’ affective state, including frustration from pain and feelings of sadness, depression, or anxiety [9,10]. Consequently, there are significant gaps in achieving an optimal balance between game challenge and emotional engagement.

One approach to improving interaction between patients and serious games is affective computing, a type of human-computer interaction that enables systems to recognize and adapt to users’ affective states [11]. Evidence suggests that dynamic adaptation based on affective states in serious games can enhance rehabilitation outcomes by improving motivation, performance, and treatment adherence [12]. Additionally, affective computing offers real-time diagnostic capabilities that allow for personalized adjustments in gameplay in response to detected emotional cues [13].

### Theoretical Background

#### Affective Computing

Picard [11] introduced the term “affective computing,” defining it as “computing that relates to, arises from, or deliberately influences emotion.” This definition highlights 3 key elements: (1) recognition, for detecting and classifying affective states; (2) expression, for displaying affective states through interaction media such as avatars; and (3) influencing affect, by using emotional models to guide behavior and decision-making.

Affective computing integrates input recognition to create an affective loop, a dynamic process in which the user’s emotional states and the system’s responses continuously interact through perception, interpretation, and emotional feedback [14]. Ideally, the system should not only detect or model emotions but also actively influence the user’s affective experience, forming a closed loop in which the user’s emotional state shapes the system’s behavior and, in turn, the user’s emotional experience.

Affective computing enables a more interactive and personalized experience by considering users’ affective states. Several studies have focused on patient care. For example, Maj et al [15] developed a system that enhances care for individuals with chronic conditions or those in rehabilitation by continuously monitoring their affective state. This system also provides real-time affective feedback and virtual companionship through a personal assistant module, offering emotional support and helping to reduce loneliness and isolation. The benefits of affective computing have been demonstrated in digital mental health interventions [16,17], adaptive systems that adjust difficulty based on users’ cognitive or emotional states [18], human-robot interactions with real-time emotion recognition and task adaptation [19], and adaptive systems with real-time feedback [20]. When combined with serious games, affective computing allows adaptive systems to modify game parameters in response to users’ emotions, such as reducing difficulty during stress or sadness and increasing it when users are motivated or engaged.

#### Emotion Recognition Techniques

According to Hurst et al [21], affective recognition techniques can be categorized into invisible and visible inputs. Invisible inputs detect physiological signals imperceptible to the human eye, such as electrodermal activity or heart rate variability. Conversely, visible inputs include observable behaviors such as facial expressions and body movements.

Techniques based on invisible inputs generally use sensors that are placed on a part of the body. However, these techniques may present some challenges, such as mild discomfort for the individual when sensors are attached to the body. The most commonly used techniques are based on electrocardiogram and electroencephalogram (EEG). The electrocardiogram is an electrical tracing of the heart and is recorded noninvasively by electrodes placed on the body. EEG is a test that measures the brain’s electrical activity by placing electrodes, which are small metal discs, on the scalp.

Techniques based on visible inputs do not require the user to be in direct contact with a sensor. Examples include facial expression analysis, user behavior monitoring, and gesture recognition. Facial expression techniques gather information about affective states from facial cues, even at rest. As a result, many studies have explored the link between facial movements and affective states [22]. User behavior techniques infer emotional states based on interaction with the system, including task performance, time spent on tasks, number of clicks, and mouse movement [23]. Gesture recognition techniques infer emotions from motor responses such as body movement, hand movement, or finger pressure [24].

Emotions are difficult to identify. According to Gonçalves et al [25], emotions are triggered by interrelated changes in 5 components: cognitive appraisals, behavioral tendencies, motor expressions, physiological reactions, and subjective feelings. To detect emotions efficiently, it is necessary to use a combination of tests that include as many components as possible. Therefore, no single emotion recognition technique is sufficient on its own.

#### Models for Classifying Affective States

The classification of affective states varies across studies; however, 2 principal models are predominantly used in affective computing: the discrete emotion model and the dimensional emotion model [26].

One discrete model is that proposed by Ekman [27], which conceptualizes emotions as discrete, measurable, and linked to physiological responses. Ekman identified 6 basic emotions considered to be universal: happiness, sadness, anger, fear, surprise, and disgust. In contrast, Plutchik [28] proposed a model comprising 8 basic emotions: joy, fear, surprise, trust, sadness, anger, disgust, and anticipation, organized in a wheel-like structure. This model suggests that more intense emotions are located at the center of the wheel, while less intense emotions are found at the periphery.

**Figure 1.**
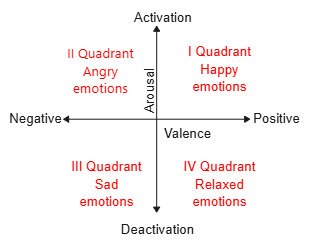
Illustration of the valence-arousal model proposed by Russell [30].

Dimensional models were introduced to overcome the limitations of discrete models by acknowledging the complexity and fluidity of emotional experiences [29-32]. For instance, Russell [30] developed the circumplex model based on valence and arousal to represent complex emotions, in which emotions are classified within quadrants. The model proposes a 2-axis plane, where the x-axis represents valence, indicating the degree of pleasure or displeasure (positive or negative), and the y-axis represents arousal, symbolizing the level of activation or deactivation. The first quadrant represents emotions associated with happiness, the second encompasses emotions related to anger, the third includes sadness, and the fourth is associated with calm emotions. The circumplex model is illustrated in Figure 1.

### Related Works

Recent advances in affective computing focus on developing techniques to detect affective states and integrating real-time affective feedback within adaptive systems. This is especially relevant in digital health, human-computer interaction, and intelligent rehabilitation. For instance, one study shows that emotion-sensitive adaptive approaches can promote task persistence and enhance in-game performance [33]. Another review finds that machine learning models support real-time personalization of psychological treatments by including behavioral and affective signals [34]. A systematic review by Leong et al [35] focused on facial emotion recognition, evaluating classification techniques and technologies used, while emphasizing the increasing use of deep learning. Despite its contributions, the review is limited to 2 emotion recognition techniques and does not examine adaptive mechanisms within the software.

These studies do not focus specifically on physical rehabilitation, where emotional engagement is essential for treatment adherence. Therefore, there is a critical need to explore how affective computing can support empathetic interactions in rehabilitation contexts, through either the adaptation of gameplay elements or the application of novel emotion detection techniques.

### Objective

This paper presents a scoping review of affective computing technologies, techniques, and methods used in serious games for physical rehabilitation, following the PRISMA (Preferred Reporting Items for Systematic Reviews and Meta-Analyses) guidelines. It offers a comprehensive overview of the current research and highlights key gaps for future developments in this field.

### Research Questions

To assess the current landscape of research in affective computing within serious games for physical rehabilitation, the following research questions were formulated:

RQ1: What theoretical approaches to emotion were applied in the studies, and which emotions were recognized?

RQ2: What techniques, including sensors and algorithms, were used for emotion recognition?

RQ3: Which game elements were adapted in response to affective state detection?

RQ4: What specific hardware was used to support the rehabilitation process?

## Methods

### Study Design

Given the objective of providing a broad overview of the literature and the diverse nature of the research questions, a scoping review was determined to be the most appropriate methodological approach. Scoping reviews are particularly suitable when the volume of publications or the heterogeneity of the topic precludes more focused methodologies such as systematic reviews [36].

This study adhered to the reporting standards outlined in the PRISMA protocols extension for scoping reviews [37] (Checklist 1).

### Inclusion and Exclusion Criteria

Due to the limited available literature on this particular topic and to ensure the inclusion of all relevant contributions, studies with varied designs and outcome measures were considered. The inclusion criteria were as follows: papers published in English, regardless of publication date; studies that used affective computing within a serious game for physical rehabilitation; and studies that involved at least 1 patient in the evaluation process. Participants in the included studies were required to have a condition necessitating physical rehabilitation.

Review papers (eg, systematic reviews, scoping reviews, and literature reviews) were excluded to focus on primary empirical evidence.

### Information Sources and Search Strategy

A comprehensive literature search was carried out in March 2024 and updated in February 2025 across the following databases: Google Scholar, PubMed, ScienceDirect, IEEE Xplore, ACM Digital Library, SpringerLink, and PEDro (Physiotherapy Evidence Database). The search strategy used the following keywords: “(affective OR emotional) AND serious (game OR games) AND rehabilitation.” The number of results retrieved from each database is presented in Table 1.

**Table 1.** Databases used.

Database	Results, n
Google Scholar	4370
PubMed	38
ScienceDirect	453
IEEE Xplore	5
ACM Digital Library	41
SpringerLink	354
PEDro	32

### Selection of Sources of Evidence

Three authors independently reviewed the records obtained through the search. Duplicate entries were removed using Mendeley Desktop (version 2.122.0) Reference Manager. Studies that met the inclusion criteria based on the title and abstract were retrieved in full text for further evaluation. Any disagreements among the reviewers were resolved by a fourth reviewer, who made the final decision.

### Data Charting Process and Data Items

Once the primary papers were identified, each was analyzed by at least 2 reviewers. These reviewers were responsible for extracting essential information from each study. Subsequently, the reviewers met to reach a consensus on the extracted data. The following data items were identified per paper: study, citation, affective computing technique, sensor or device used to detect affective states, population characteristics (age and sex), type of disease, type of rehabilitation, game elements adapted in response to affective states, affective states detected, machine learning algorithms or software used for affective state detection, and theoretical approaches to affective states.

### Assessment of the Quality of the Included Studies

The methodological quality of the selected studies was evaluated using the QUADAS-2 (Quality Assessment of Diagnostic Accuracy Studies) tool [38], which assesses 5 domains: sample selection, index test, reference standard, flow, and timing (Multimedia Appendix 1). Risk of bias and overall study quality were reviewed by 2 authors. Any disagreements in the assessment process were resolved through discussion and consensus within the research team. Question 1 in domain 3 was modified to require at least 2 tests to validate the target condition. The question was modified because, although there is no gold standard for validating emotions, Gonçalves et al [25] suggest that 5 components should be considered: cognitive appraisals, behavioral tendencies, motor expression, physiological reactions, and subjective feelings. Although validating the emotion using the 5 components would be more effective, it is also more complex. For this reason, it was expected that at least 2 tests would be used.

### Synthesis of Results

A formal narrative synthesis was conducted to evaluate the influence of affective computing on the rehabilitation process.

## Results

### Selection and Characteristics of Sources of Evidence

**Figure 2.**
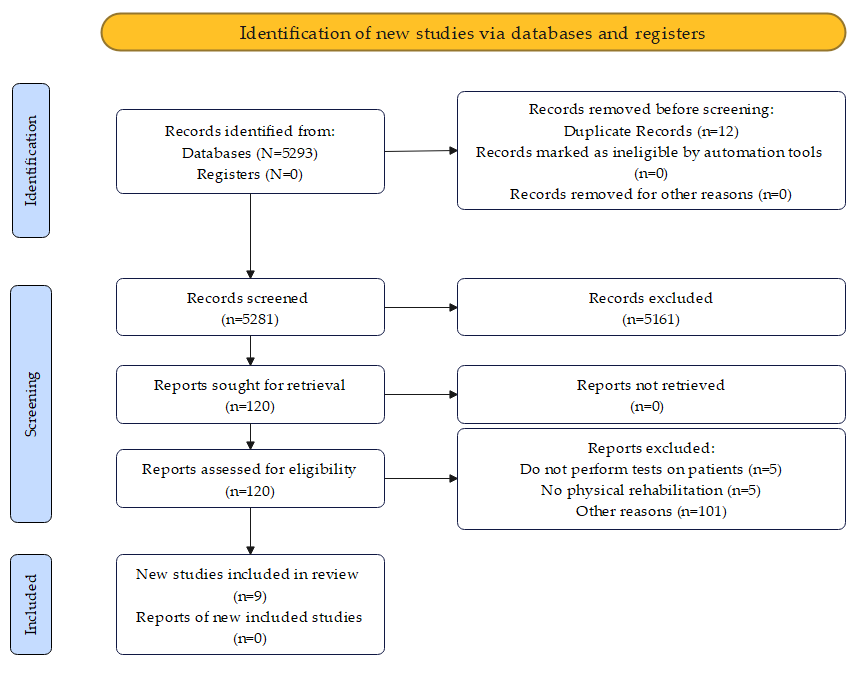
PRISMA (Preferred Reporting Items for Systematic Reviews and Meta-Analyses) flow diagram illustrating the selection process of the included studies.

A total of 5293 records were identified through database searching. After removing 12 duplicate records, 5281 records were screened based on title and abstract, of which 5161 were excluded. As a result of this screening process, the full texts of 120 reports were sought for retrieval, all of which were successfully obtained and assessed for eligibility. Of these, 111 reports were excluded for various reasons, including interventions involving healthy individuals, games not involving physical rehabilitation, or review papers. Finally, 9 studies were included in the systematic review (Figure 2).

### Results From Individual Sources of Evidence

#### Analysis of the Research Questions

RQ1: What theoretical approaches to emotion were applied in the studies, and which emotions were recognized?

As shown in Table 2, 3 of the analyzed studies used the dimensional model [39-41], while the others did not specify the emotion model applied. The authors noted that affective states were classified as discrete (eg, pain and fatigue), enabling researchers to address these states as events requiring immediate intervention by therapists or the rehabilitation systems. Additionally, psychophysiological states were identified as having a direct impact on patients’ motor performance.

Overall, the studies address affective states with direct clinical relevance to patient progress: (1) pain and anxiety are the main barriers to motor recovery, as they may lead patients to discontinue therapy; (2) physical and psychological fatigue are critical to monitor to prevent overexertion and frustration; and (3) engagement and motivation are essential for long-term treatment adherence. Studies such as those by Rivas et al [42,43] also highlight the interdependence among these states. For example, pain often coexists with anxiety, while engagement rarely occurs alongside extreme fatigue.

RQ2: What techniques, including sensors and algorithms, were used for emotion recognition?

Analysis of the 9 studies indicates that affective computing in virtual rehabilitation uses sensor-based techniques, artificial intelligence (AI) algorithms and models, and data integration to assess patients’ affective states.

#### Sensor-Based Techniques

The reviewed studies classify sensors into 3 primary categories based on their nature and degree of intrusiveness.

- Movement-based and force-based systems: A notable trend in the field is the adoption of low-cost devices that integrate 3D position and pressure sensors to capture hand location and grip strength [42-46]. This nonintrusive approach allows affective states to be inferred directly from rehabilitation platform data, eliminating the need for additional sensors on the patient.
- Physiological and neurophysiological signals: Neurophysiological signals, especially EEG, are used to capture brain activity for the direct interpretation of affective states. Some approaches classify basic emotional valence (positive or negative) [41], while others use wearable devices to monitor complex states such as engagement, boredom, or frustration. These methods are also applied in areas such as gait rehabilitation [40].
- Computer vision and posture capture: The application of visual sensors has evolved from specific limb tracking, such as hand position analysis by Funabashi et al [47], to more robust multimodal approaches. Recent studies integrate conventional webcams for simultaneous analysis of facial expressions and body posture [42-45]. This integration enables a more comprehensive, contextually informed interpretation of the user’s affective state.

**Table 2.** Summary of the studies included in the review.

Study	Affective computing technique	Sensor used	Type of study	Elements adapted in video game	Patient population	Age and sex	Disease	Rehabilitation	Affective states detected	Machine learning algorithms or software used to detect affective states	Emotion theoretical approaches
Funabashi et al [47]	Facial expressions	Webcam	Proof-of-concept	Not available	4	Not available	Stroke	Upper limb	Engagement, valence	Affectiva SDK^a^	Not available
Rivas et al [44]	Facial expressions, gesture recognition	Webcam, sensor to obtain 3D coordinates, and pressure sensor	Feasibility pilot	Not available	2	Not available	Stroke	Upper limb	Tiredness, tension, pain, and satisfaction	Linear support vector machines	Not available
Tironi et al [39]^b^	User behavior	Microsoft Kinect	Proof-of-concept	Facial expressions, movements, and gestures of the avatar	1	Not available	Posture problem	Postural	Valence and arousal	Patient’s game performances and textual inputs	Core affect, Russell [31]
Rodriguez et al [40]	Electroencephalogram	Emotiv Insight, Affect-Driven Self-Adaptive System (ADAS)	Preliminary study	Not available	8	Range 5‐13 y; 4 females and 4 males	Gait-related impairment	Lower limb	Engagement, boredom, excitement, frustration, and meditation	Emotive SDK, ADAS software	Pleasure-arousal-dominance dimensional model, Mehrabian [29]
Rivas et al [45]	Facial expressions, gesture recognition	Webcam, sensor to obtain 3D coordinates, and pressure sensor	Feasibility study	Not available	5	Range 41‐67 y; 4 females and 1 male	Stroke	Upper limb	Tiredness, anxiety, pain, and motivation	Fusion Semi-Naive Bayesian (FSNB) classifier, Multiresolution Semi-Naive Bayesian (MSNB) classifier	Not available
Hou and Sourina [41]^b^	Electroencephalogram	Emotiv Insight	Proof-of-concept	Background music, visual adaptation, game difficulty	1	69 years; gender not specified	Stroke	Upper limb	Positive and negative	Real-time fractal dimension-based valence-level recognition algorithm	Valence-arousal-dominance emotion model, Russell and Mehrabian [32]
Rivas et al [46]	Gesture recognition	Webcam, sensor to obtain 3D coordinates, and pressure sensor	Feasibility study	Not available	5	Range 41‐67 y; 4 females and 1 male	Stroke	Upper limb	Tiredness, anxiety, pain, and engagement	MSNB classifier	Not available
Rivas et al [42]	Facial expressions, gesture recognition	Webcam, sensor to obtain 3D coordinates, and pressure sensor	Methodological and validation study	Not available	11	Range 41‐76 y; 4 females and 7 males	Stroke	Upper limb	Tiredness, anxiety, pain, and engagement	FSNB classifier, Circular Classifier Chain (CCC)	Not available
Rivas et al [43]	Facial expressions, gesture recognition	Webcam, sensor to obtain 3D coordinates and pressure sensor	Methodological and validation study	Not available	5	Range 41‐67 y; 4 females and 1 male	Stroke	Upper limb	Tiredness, anxiety, pain, and engagement	Semi-Naive Bayesian classifier, FSNB classifier, CCC	Not available

a SDK: software development kit.

b Games perform dynamic adaptation.

#### Algorithms and AI Models

The evolution of algorithms in this field has progressed from simple classifiers to architectures capable of addressing the complexity and interdependence of affective states: (1) probabilistic and Bayesian models, such as Semi-Naive Bayesian classifiers, have been used by Rivas et al [42,43,45,46] to support patients who have experienced stroke during upper-limb rehabilitation exercises; (2) dependency models, including multilabel approaches such as Circular Classifier Chains, have been implemented to simultaneously recognize multiple affective states (eg, pain, anxiety, and fatigue) [42,43]. These algorithms are critical because they account for the correlation among affective states; (3) fuzzy logic has been used in the Emphatic Virtual Caregiver system developed by Tironi et al [39], which applies fuzzy inference to process patient performance and affective state, thereby enabling smooth, natural transitions in the virtual caregiver’s responses; (4) traditional machine learning techniques, such as support vector machines [44], have been documented in preliminary stages for classifying affective states, including tiredness, tension, pain, and satisfaction, based on descriptors of 3D hand movement and finger pressure.

#### Data Integration and Fusion

A trend in the papers is the adoption of multiple techniques for detecting affective states. Rather than relying on a single sensor, the systems proposed by Rivas et al [42-45] combine data from limb movements, facial expressions, and finger pressure. This integration permits the fusion algorithm to function as a higher-level layer, weighting information from each source to generate a more robust final emotional label that is resilient to noise and variability among different motion-sensing devices.

RQ3: Which game elements were adapted in response to affective state detection?

This research question seeks to determine which elements of serious games are adapted or modified in response to users’ affective states. Four categories of adaptation were identified: auditory, visual, difficulty, and no adaptation.

#### Auditory Adaptation

Auditory adaptation is primarily used to regulate negative emotions and increase user engagement in serious games for rehabilitation, generally through background music and sound effects. This approach is frequently implemented when users display negative emotions despite correct task performance, indicating that auditory feedback serves as a compensatory mechanism to improve mood without changing the task structure, as observed in Hou and Sourina [41].

#### Visual Adaptation

Visual adaptation is a flexible approach to addressing users’ affective states by modifying graphical user interface elements, such as avatars, environments, lighting, and in-game objects. These adaptations occur at expressive and environmental levels, involving modifications in facial expressions, gestures, object configurations, color schemes, and visual complexity.

At the expressive level, systems dynamically adjust avatar behavior to reflect users’ emotional states, enabling empathetic responses through facial expressions and body movements, as implemented in Tironi et al [39]. At the environmental level, visual properties of the game—such as object shapes, colors, and scene configuration—are modified in response to users’ affective states, particularly when negative emotions are detected despite adequate performance, as reported by Hou and Sourina [41].

Overall, visual adaptation works as both a reactive mechanism and a tool for modulating the user’s emotional experience. This dual capacity highlights its potential to reflect and influence affective states in rehabilitation contexts.

#### Difficulty Adaptation

Difficulty adaptation is based on flow theory [48], which highlights the need to balance task challenge with the user’s ability to maintain engagement. This balance is especially important in rehabilitation, where tasks must be adjusted to avoid both frustration and disengagement.

Hou and Sourina [41] found that task difficulty increases when users perform successfully and show positive affect. If negative affect is present, task difficulty remains unchanged. This asymmetry indicates a conservative adaptation strategy, prioritizing performance-driven progression while minimizing risks linked to lowering task demands.

#### No Adaptation

Unlike studies that incorporate adaptive mechanisms, some investigations do not adjust game elements in response to emotional recognition. In these instances, affective computing serves primarily analytical functions and does not directly influence system dynamics.

For example, Funabashi et al [47] evaluate user engagement and affective responses without altering gameplay. Similarly, Rodriguez et al [40] use electroencephalographic signals to identify emotional states, but do not use this information to modify game behavior.

Other studies use various machine learning algorithms for emotion recognition, but these data are not used to adjust game elements in real time. However, the authors agree that identifying states such as pain, anxiety, or fatigue aims to personalize rehabilitation tasks.

Specifically, Rivas et al [42-46] focus on improving the accuracy of affective state inference and propose involving adaptive mechanisms in future research. These mechanisms include adjusting difficulty levels and modifying the virtual environment to maintain a safe, motivating, and needs-based therapeutic context for patients.

The results show that affective computing in rehabilitation remains in a validation phase focused on identifying affective states. Analysis of the studies indicates that researchers have prioritized accurate identification of patients’ affective states, such as stress, fatigue, or anxiety, before delegating clinical decision-making to software. The limited implementation of adaptation implies a lack of consensus regarding appropriate system responses to detected emotions, such as whether the system should halt or adjust task difficulty when negative emotions are identified.

RQ4: What specific hardware was used to support the rehabilitation process?

Analysis of the 9 included studies identifies a diverse technological infrastructure designed to support therapeutic exercise and enhance interactivity. Hardware used in physical rehabilitation can be categorized into 3 functional groups: nonintrusive motion capture sensors, haptic devices, and visualization and immersion hardware.

#### Nonintrusive Motion Capture Sensors

The reviewed studies demonstrate the use of nonintrusive technologies to capture movement during rehabilitation, allowing natural user interaction without invasive wearable devices. Rivas et al [42-46] use 3D position sensors to record therapeutic movements and supplement this data with webcams to capture facial expressions and gestures, enabling richer analysis through multiple data sources.

Similarly, Funabashi et al [47] use the Microsoft Kinect for Xbox One, which uses RGB-D (red, green, blue, and depth) technology to track and evaluate body movement. This device supports rehabilitation therapies by enabling patients to manipulate virtual objects with hand movements, fostering intuitive interaction within gamified therapeutic settings.

Collectively, these approaches demonstrate a trend toward the incorporation of accessible and nonintrusive sensors that enable accurate motion capture and integration with additional modalities. This integration supports a more comprehensive assessment of patients’ performance in rehabilitation settings.

#### Haptic Devices

The literature highlights the use of specialized hardware to facilitate user interaction with rehabilitation environments. These technologies vary according to therapeutic objectives, ranging from high-precision haptic devices such as the Novint Falcon for controlling serious games [41] to advanced robotic assistance like the *Lokomat*. The *Lokomat* enables lower-limb rehabilitation by integrating body-weight support and robotic legs with a treadmill [40]. Additionally, customized interfaces have been developed, including sensorized levers designed to quantify grip strength and finger pressure during exercise [42-45].

#### Visualization and Immersion Hardware

Advanced visualization systems, such as the *Oculus Go*, enhance immersion and realism in rehabilitation environments. As documented by Rodriguez et al [40], these devices provide high-fidelity 3D visuals, increasing patients’ sense of presence, which is critical for engagement and motivation during therapy.

### Synthesis of Results

The affective states detected across the studies included engagement, valence, tension, pain, satisfaction, happiness, sadness, anger, surprise, boredom, excitement, frustration, and meditation. The most frequently recognized emotions were engagement, pain, tiredness, and anxiety. Only 3 studies used theoretical frameworks of emotion. The most commonly used affective computing techniques were facial expression analysis and gesture recognition, while user behavior analysis was the least used technique. Merely 2 studies adapted serious game elements based on the affective states of users. The adaptive elements identified included modifications to background music, visual interface parameters, game difficulty, and avatar facial expressions, movements, and gestures.

### Quality Assessment

Quality assessment using the QUADAS-2 tool indicated that 88% (8/9) of the included studies exhibited a high risk of bias in the patient selection category, mainly due to insufficient descriptions of recruitment procedures. Neither random nor consecutive recruitment was reported, which substantially increases the risk of selection bias. In the index test category, all studies (n=9, 100%) clearly defined the algorithms or techniques used for detecting affective states. For the reference standard, although no gold standard exists in affective computing, it is recommended to use multiple emotion recognition methods to improve accuracy [49]. However, 88% (8/9) of the studies did not use appropriate methods to assess reliability or validate their findings. In the flow and timing domain, 33% (3/9) of the studies demonstrated a high risk of bias due to the lack of explanation of the number of sessions and the failure to report participant dropout rates.

**Figure 3.**
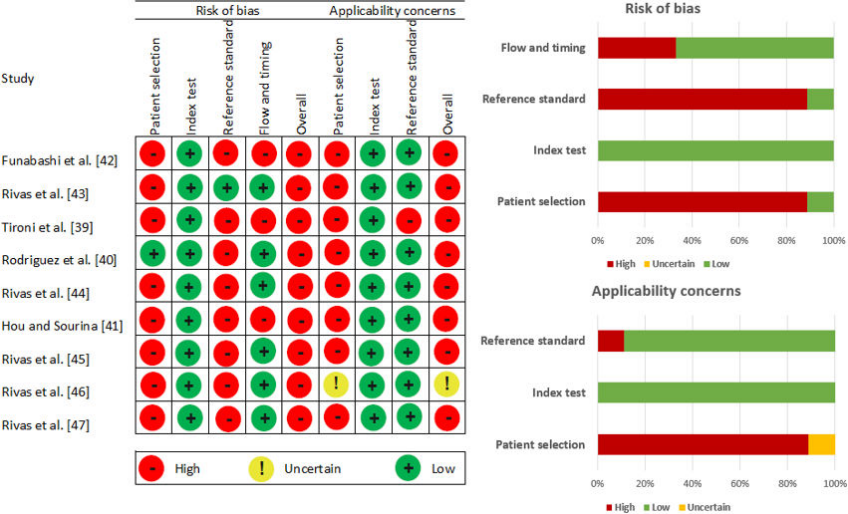
Risk of bias assessment of the included studies using the QUADAS-2 (Quality Assessment of Diagnostic Accuracy Studies) tool [39-47].

Regarding applicability, 88% (8/9) of the studies showed a high risk of bias in patient selection due to small sample sizes, limiting the generalizability of the findings. Additionally, patient recruitment from a single center further restricts the representativeness of the results for the wider patient population with varying severity levels. All studies presented low applicability concerns for the index test, as the techniques or algorithms used were clearly described. For the reference standard, 88% (8/9) of the studies exhibited low applicability concerns because they relied on clinical experts to validate emotions or used at least 2 emotion identification techniques, thereby ensuring that the system measures clinically relevant outcomes. Figure 3 illustrates the quality assessment outcomes.

## Discussion

### Implications for Clinical Rehabilitation

Given the critical role that affective states play in the rehabilitation process, this scoping review provides a comprehensive examination of the application of affective computing techniques within serious games designed for physical rehabilitation.

Studies involving healthy participants were excluded to ensure the analysis was relevant to clinical contexts. Patients frequently experience emotions such as anxiety, depression, and fear during rehabilitation. Thus, it is crucial to assess whether recognizing and adapting to these affective states can improve therapeutic outcomes. As a result, only 9 studies were included in the final synthesis. This highlights an important opportunity for future research to design studies involving patient populations where emotional state monitoring is integrated into the rehabilitation process.

Analysis of the 9 studies shows clear differences among techniques, each with specific implications for rehabilitation: (1) gestures and motion analysis, used by Rivas et al [42-46] and Funabashi et al [47], represent the predominant technique. The main advantage is its nonintrusive nature, which uses sensors already required for therapy to measure variables such as hand position and finger pressure. This characteristic makes it suitable for routine clinical practice, as it does not increase the sensory burden on patients, although its inference capability may be less direct than that of other techniques; (2) facial expression analysis, applied in multimodal approaches by Rivas et al [42-46], helps detect states such as satisfaction or pain. However, its efficacy can be compromised when patient expressions are altered, for example, throughout intense physical exercise, which may lead to misinterpretation by monitoring algorithms; (3) EEG, as implemented by Hou and Sourina [41] and Rodriguez et al [40], directly measures brain activity and allows precise detection of affective states such as emotional valence. However, EEG used in serious games for physical rehabilitation is limited because electrodes can be intrusive for patients, and the technique is highly sensitive to noise from muscle movements. This sensitivity may limit its use during dynamic exercise in clinical settings.

A common limitation across the reviewed literature is the small sample size, which raises concerns regarding the generalizability and reliability of the reported findings. Future studies should aim for larger, more representative samples. Methodological guidelines, such as those proposed by Fritz et al [50], should be used for sample size calculation. Additionally, the reasons for participant dropout in physical rehabilitation interventions should be thoroughly documented, as this information can help inform and improve the design of future studies.

Research involving human participants is one of the most highly regulated areas in scientific inquiry, particularly regarding ethical standards. Data obtained from affective computing studies may be used for various secondary purposes, including video labeling, database inclusion, or training AI models. Consequently, adherence to ethical frameworks, such as those established by the American Psychological Association [51], is essential. Among the studies reviewed, only Rodriguez et al [40] explicitly reported compliance with the Declaration of Helsinki, received ethics committee approval, ensured data protection, and obtained explicit consent for video and photographic recordings. In contrast, several studies [42-44,46] merely acknowledged consent for video recordings, and others did not mention compliance with ethical guidelines at all, raising serious concerns regarding the ethical implications of affective computing applications. Clinical data privacy, especially in pediatric populations, must be a priority. Ensuring data confidentiality in storage, transmission, and usage is essential.

Regarding emotion theory, there is a lack of standardization, which makes direct comparisons between systems difficult; most studies did not justify their choice of emotion model. Among those that did [39-41], the dimensional approach to emotion was the most frequently adopted. This approach appears sufficient for many serious game applications, as determining whether a patient’s affective state is generally positive or negative may be adequate for making gameplay adaptations such as altering difficulty or modifying environmental elements.

Another issue identified is the classification of pain as an emotion, which was reported in 50% (4/8) of the studies [42-46]. However, existing literature [52,53] suggests that emotions like depression, anxiety, and anger often emerge as psychological responses to pain rather than being emotions themselves. Therefore, classifying pain as an emotion may be conceptually inaccurate, highlighting the need for a more nuanced and consistent emotional taxonomy in future research.

However, detecting pain and fatigue (physical and psychological) is essential in rehabilitation, as these are major barriers to motor recovery. Pain may cause anxiety and fear of movement [43,46]. Nonintrusive pain detection allows systems to adjust challenges in real time, prevent secondary injuries, and maintain patient safety, which can improve engagement.

By detecting patient fatigue, the system can recommend breaks, adjust exercises, or lower the difficulty to maintain high motor quality and enhance the rehabilitation process.

Clinical applicability is a further consideration when translating research into practice. When selecting data acquisition devices, factors such as cost, precision, portability, patient comfort, and mobility should be considered. Some devices, such as standard webcams [42-47], are easily accessible and cost-effective, while others, like the Emotiv Insight [40,41], require a more substantial investment.

Patient characteristics should also inform the choice of emotion recognition techniques. For instance, in pediatric rehabilitation, noninvasive methods should be prioritized to accommodate children’s natural restlessness and their need for freedom of movement. In studies using facial expression analysis, factors such as ambient lighting, camera specifications, and the distance between the patient and the device can influence outcomes. However, these conditions were not reported in the included studies, and such factors must be considered when selecting or developing software libraries or AI algorithms.

An emerging trend identified is the integration of machine learning techniques. Findings suggest that combining multiple machine learning methods or modifying existing classifiers, such as the one proposed by Rivas et al [45], can enhance emotion recognition accuracy in virtual rehabilitation settings. These findings point to promising future directions for the integration of advanced AI methods in affective computing research.

Due to the variability in study designs and small sample sizes, the findings of this scoping review must be interpreted with caution. While affective computing appears to be a feasible strategy for enabling emotion-sensitive adaptations in serious games, the evidence for its clinical efficacy remains inconclusive. Accordingly, future research should adopt more rigorous methodologies, including larger patient samples, standardized protocols, and validated outcome measures, such as the Fugl-Meyer Assessment or Berg Balance Scale, to better assess the therapeutic impact.

Zhao et al [49] suggest that using multiple affective state recognition techniques can improve classification accuracy and the effectiveness of affective adaptations. However, only 4 of the studies [42-45] used more than 1 recognition method, with gesture and facial expression analysis being the most common combination. It is therefore recommended that future studies use at least 2 complementary techniques, selected based on their noninvasiveness and their ability to preserve patient mobility during rehabilitation.

User behavior analysis was the least explored approach, despite its potential for nonintrusive emotion detection. In this review, only Tironi et al [39] incorporated this technique, and its effectiveness remains to be validated. Future research should consider the application and validation of this approach in clinical rehabilitation settings.

The field remains underdeveloped, with most studies focused on technical validation or proof-of-concept, rather than rigorous experimental designs. The lack of randomized controlled trials is a significant limitation, preventing affective computing from being established as an evidence-based intervention. As a result, these technologies are still considered experimental. Future research should use multicenter designs with statistically significant samples and control groups to validate the long-term clinical value of affective computing.

Analysis of the 9 studies highlights a major limitation in translating affective state recognition into functional application: adaptive approaches are infrequently implemented, with only 2 out of 9 studies [39,41] using a functional closed-loop system that detects emotion and adapts therapy accordingly. Three primary barriers account for this limitation: (1) clinical safety, as incorrect adaptations based on false positives may delay patient progress or compromise treatment effectiveness, for example, if physical effort is misinterpreted as pain and exercise intensity is reduced; (2) technical complexity, since real-time integration of patient signal processing with game engine logic requires considerable coordination among components such as game mechanics and hardware; and (3) the absence of theoretical intervention models, as current psychological models define affective states but do not provide protocols for automatic responses in affect-based rehabilitation systems [13]. As a result, researchers frequently use open-loop systems, in which software monitors the patient, but a human expert, such as the therapist, ultimately adjusts the therapy.

The application of the QUADAS-2 quality assessment tool revealed a high risk of bias in study participant selection, as most studies have small sample sizes. A high risk of bias was also found in the reference standard, since emotions were labeled through expert observation of video recordings. Subjectivity raises concerns that the emotion-detection algorithm, or index test, may simply replicate the biases of human observers. A moderate risk was observed regarding flow and timing, as several studies did not specify the testing interval. Considerable concerns also exist regarding the applicability of patient selection, since studies are often conducted in controlled research laboratories that may not accurately reflect real clinical practice, given patient variability as well as environmental factors such as movement and external noise.

The combination of serious games with affective computing facilitates the identification of states that may hinder rehabilitation, such as pain, fatigue, or anxiety. Failure to deal with these barriers can result in treatment abandonment or injuries due to overexertion in the short or medium term. Nonintrusive detection of pain or fatigue has direct implications for patient safety. In conventional clinical practice, therapists rely on patient self-reports, which may be biased by a desire to please health care professionals, communication difficulties, or embarrassment. Automated detection systems can support health care professionals in making more informed rehabilitation decisions. Affective serious games may serve as objective safety monitors, enabling the system to reduce challenge intensity before patients reach risk thresholds. Additionally, the inclusion of empathetic virtual agents offers a clinical strategy to address loneliness or depression associated with disability. Furthermore, integrating affective computing improves the feasibility of home-based therapy systems by delivering health care providers and family members with alerts regarding patient progress and emotional health.

### Limitations

This scoping review presents several limitations. Although systematic search strategies were used to identify relevant studies across major electronic databases using carefully selected keywords, terminological heterogeneity within the literature may have resulted in the inadvertent omission of pertinent studies.

A major limitation of this review is the small number of included studies (n=9), which limits the ability to draw robust generalizations about the implementation of affective computing in serious games for physical rehabilitation. As a result, the identified trends should be viewed as preliminary, indicating that the integration of clinical rehabilitation and affective computing is still in an early stage of development.

The exclusion of gray literature further limits the scope of this review. By focusing exclusively on peer-reviewed studies, the review ensures a consistent standard of quality, coherence, and reliability. In contrast, gray literature often lacks external validation and standardized reporting structures, which increases the risk of methodological errors and interferes with the assessment of bias, sample representativeness, and the robustness of conclusions.

Another limitation is the absence of searches in languages other than English. All included studies are published in English in high-impact journals or specialized conferences, such as IEEE. This approach may exclude relevant research in other languages and obscure relevant regional developments.

Finally, the findings of this review should be interpreted in light of the methodological limitations of the included studies. First, most studies use small sample sizes, typically 1 to 11 participants. Second, there is also significant selection bias, as many studies rely on samples from specific institutions and often do not clearly describe the patient selection process. Third, most studies focus only on inferring and classifying affective states without implementing adaptive mechanisms to complete the functional loop.

### Conclusions

This scoping review presents an updated overview of the state of research on the application of affective computing in serious games for physical rehabilitation. The studies analyzed identify various emotional states in patients undergoing rehabilitation through serious games. These papers use machine learning algorithms or commercial software development kits for emotion detection through at least 1 modality, such as facial expressions, EEG, gesture recognition, or user behavior.

The study shows that applying affective computing techniques to serious games for physical rehabilitation offers a promising complement to traditional rehabilitation approaches for various types of disabilities, incorporating patients’ affective states into the design and adaptation of these games. While affective classification methods have advanced, closing the affective loop through automated interventions remains rare in serious game-based rehabilitation systems. Moreover, the limited availability of primary sources weakens the conclusions regarding adherence and functional performance. Consequently, there is a strong need for multicenter studies with rigorous methodologies to develop systems with a greater impact on rehabilitation.

Therefore, the current evidence base for affective computing in rehabilitation through serious games is technically advanced, yet methodologically fragile. Existing studies remain in a model exploration phase, demonstrating the feasibility of emotion detection but lacking reliability at scale due to selection biases and uncertainties regarding real-life applicability.

Future research should consider the following key aspects: (1) methodological design, including a clear description of the theoretical approaches to emotion, patient selection criteria and characteristics, and a detailed description of the index tests and reference tests used; (2) the adaptation of serious game elements based on the patient’s affective state; (3) use of validated motor function assessment scales to evaluate improvements in the rehabilitation treatment; and (4) promotion of multidisciplinary collaboration among experts in affective computing, physical therapists, and mental health professionals such as psychologists or psychiatrists.

We hope this study serves as a foundation for future investigations seeking to advance physical rehabilitation by integrating affective computing technologies.

## Supplementary material

10.2196/81344 Multimedia Appendix 1 Questions of the QUADAS-2 tool.

10.2196/81344 Checklist 1 PRISMA-ScR checklist.

## Abbreviations

AI: artificial intelligence
EEG: electroencephalogram
PRISMA: Preferred Reporting Items for Systematic Reviews and Meta-Analysis
PRISMA-ScR: Preferred Reporting Items for Systematic Reviews and Meta-Analysis–Extension for Scoping Review
QUADAS: Quality Assessment of Diagnostic Accuracy Studies
RGB-D: red, green, blue, and depth
