# Supplementary material for: Affective Computing in Serious Games for Physical Rehabilitation: Scoping Review
Source: JMIR Rehabil Assist Technol. 2026 Jul 2;13:e81344. doi: 10.2196/81344 (PMC13325924; doi:10.2196/81344)
Supplement: Multimedia Appendix 1 [file rehab-v13-e81344-s001.pdf]

Multimedia Appendix 2

Table 1. Questions of the QUADAS-2 Tool

Domain name	Questions
Risk of bias	
1. Patient selection	1. Was a random or consecutive sample of patients enrolled?<br>2. Was a case-control design avoided?<br>3. Did the study avoid inappropriate exclusions?
2. Index test	1. Were the index test results interpreted without knowledge of the results of the reference standard?
3.Reference standard	1. Is the reference standard likely to have accurately identified the target condition, using at least two independent assessments? *<br>2. Were the reference standard results interpreted without knowledge of the results of the index test?
4. Flow and timing	1. Were both the index test and the reference standard conducted during the same rehabilitation session? *<br>2. Did all patients receive a reference standard?<br>3. Were all patients included in the analysis?
Applicability	
1. Patient selection	1. Are there concerns that the included patients and setting do not match the review question?
2. Index test	1. Are there concerns that the index test, its conduct, or its interpretation differ from the review question?
3.Reference standard	1. Are there concerns that the target condition as defined by the reference standard does not match the question?

* This element was modified.
